# Free psychology? Why psychological research is incompatible with the requirements of clockwork determinism

**DOI:** 10.3389/fpsyg.2025.1544101

**Published:** 2025-05-07

**Authors:** Stephan Lau, Roy Frederick Baumeister

**Affiliations:** ^1^Federal University of Administrative Sciences, Berlin, Germany; ^2^Department of Psychology, Faculty of Arts and Sciences, Harvard University, Cambridge, MA, United States

**Keywords:** determinism, philosophy of science, psychological science, compatibility, epistemological limits

## Abstract

This essay argues that the concept of strict causal determinism (or “clockwork determinism”), while being a powerful doctrine to reduce uncertainty, is not compatible with the way psychology does science. Specifically, we argue that psychological explanations are necessarily incomplete, that the specification and measurement of variables will always contain variance, and that psychological experiments cannot guarantee the degree of control necessary for strict deterministic relationships. Further, we argue that typical psychological causes do not fit the scale of clockwork-deterministic explanations. It is important to note that these arguments are agnostic to the question of whether clockwork determinism exists or not. Even if the universe works strictly deterministically, psychological explanations and paradigms would remain incompatible with the requirements posed by clockwork determinism. We judge this not to be of any problem for a thriving psychological science, unless (young) scientists see clockwork determinism as their primary epistemological foundation.

## Introduction

If you are thirsty and drink three glasses of water, you will no longer be thirsty. If you heat water to 100 degrees Celsius, it will boil. If you threaten someone’s personal freedom, he or she will become reactant. These statements are examples of what Doob (1988) calls “inevitability doctrines,” that is, rules (or laws) that predict what will happen. Such rules suggest the controllability of events by employing lawful prediction, thus seeming to represent classic causal determinism. The doctrine of classic causal determinism, which we term *clockwork determinism* (see Koch, 2009), consists of strict and inevitable relationships between variables and events. Clockwork determinism claims to be an epistemological foundation for much of the work in natural and social sciences (Gadenne, 2004). However, as we argue in this paper, psychology hardly fulfills the requirements of clockwork determinism irrespective of the truth of the doctrine. Psychology appears to be incompatible with clockwork-deterministic premises insofar as it continually deals with multiple possibilities of human behavior. Strong and all-encompassing “if…then” clauses are not illustrative of what makes psychological science. Psychologists typically must acknowledge degrees of uncertainty (i.e., chance, variance), which precludes unanimously true and inevitable explanations. The introductory statements above exemplify this uncertainty, because none of them is universally true: polydipsia can make you feel thirsty even after three glasses of water, the boiling point of water changes with altitude (i.e., environmental pressure), and the occurrence of reactance is contingent on a variety of boundary conditions. Thus, statements in terms of “If X then Y” most often only *seem* to express fully deterministic relationships, while actually their validity is not only limited but also leaves room for alternative outcomes.

According to Doob (1988), human beings actively pursue inevitability to reduce uncertainty (i.e., ignorance, uncontrollability). There are several doctrines (i.e., sets of beliefs, laws, or rules) that can be used for this purpose. Determinism is one of them. We argue that clockwork determinism is not a promising doctrine for psychology to use in its pursuit of reducing uncertainty, for multiple reasons, including that it is incompatible with the way science is conducted. However, a deterministic outlook on human behavior is lingering and pervasive, particularly among younger scientists, as discussions about free will and a survey of social psychology colleagues show (see Baumeister and Lau, 2024). We therefore present four straightforward arguments why, if clockwork determinism is to be taken seriously, psychological science is incompatible with its requirements, thereby making it an impractical doctrine.

Reflecting the fit of clockwork determinism to metaphysical doctrines clarifies the long-term goals of psychological science. If the aim is to locate and prove a law behind everything psychological, as classic determinism would prescribe (Gadenne, 2004), we might find ourselves in dead ends when realms of complexity are encountered where limited methods prevent looking further behind the veil. As we argue below, this dead end is not a mere possibility but rather a logical consequence given the requirements of a fully deterministic explanation of human behavior on the one hand and the methodological capacities of psychological science on the other. Alternatively, an open approach to uncertainty might be adopted that enables investigation of cognitive and behavioral processes in all their diversity but without the pretense to bind them into deterministic laws. This way we would not need to refrain from areas where uncertainty cannot be reduced further but could instead focus on the topical variety within these areas, fueled by new societal developments (e.g., investigating the mechanics of “fake news” without positing laws on those mechanics).

### What is clockwork determinism?

Many definitions of determinism are prevalent among different fields (e.g., stochastic determinism, historical determinism, adequate determinism, and so forth). We, however, refer to the notion most common and powerful when it comes to metaphysical discussions—the concept of *strict causal determinism.* As a doctrine, determinism is a very powerful approach to reduce uncertainty and replace it with inevitability (Doob, 1988). Within determinism all events are strictly bound by laws of cause-and-effect and all events do follow *inevitably* and with necessity from prior conditions, following the laws of nature (see Bunge, 1979; Gadenne, 2004; Hoefer, 2016; Koch, 2009). Thus, determinism suggests it could be possible to achieve complete knowledge (in principle) and unerringly accurate predictability of all future events. LaPlace (1820) famously embodied this idea with his all-knowing “demon.” This imaginary, super-intelligent demon knew all the laws of nature and knew the disposition of every particle in the universe at a given moment and on that basis could calculate and predict every future event with 100% accuracy. According to the Laplacian view, the universe works like “clockwork,” which has progressed on a fixed course since its start (Koch, 2009). Following Koch (2009), we therefore termed this notion *clockwork determinism*. Applied to psychology, human behavior and cognition would be fully determined by genes, upbringing, environmental stimuli, and prior events. For every behavior X, there would be a sufficient set of conditions (A, B, …) that inevitably caused X, implying the complete predictability of all human behavior. If this sounds enticing, we encourage to keep reading, as this is an unrealistic promise. Further, please note that terms like “sufficient set of conditions,” “inevitability” and “with necessity” all set exacting standards for any explanation.

For greater clarity, let us summarize our basic premise and the relationships between classic clockwork determinism and empirical science (i.e., psychology in our case). Three important claims have to be distinguished: (1) According to classic causal determinism, everything that happens is subject to strict deterministic laws, which means that based on a complete knowledge of these laws and of the initial conditions, every event (including psychological events) can be explained and predicted. (2) A goal that empirical science can set (and evidently did set in the past) is to search for strictly deterministic laws and complete deterministic explanations. We, however, argue that, (3), experience shows that no strictly deterministic laws can be found in psychology and that this is not only a provisional state but a general incompatibility between psychological science and the requirements of a fully deterministic science. We will present four arguments in detail to support claim (3: psychology is incompatible with determinism) in order to conclude that claim (2: psychology should search for deterministic laws) is a misguided undertaking. We also like to emphasize that claim (3) is not in any way contradicting claim (1) by necessity. In other words, one can be a determinist and believe in determinism to be true while acknowledging at the same time that psychology is not a fully deterministic science.^1^

## Is psychology fit to be a fully deterministic science? Arguments why this is not the case (nor a problem)

To illustrate our arguments, we resort to the well-established social psychological theory of reactance (Brehm and Brehm, 1981; Clee and Wicklund, 1980). From this theory, we can derive the simple causal hypothesis that condition A (a threat to personal freedom; here: external pressure to eliminate an option) leads to effect R (reactance; here: an increase in attractiveness of the pressured option). To have a tangible situation where we like to use this theory for deterministic explanation and prediction, let us imagine John, who is thinking about marrying his girlfriend Jane. He gets a call from his father who patronizingly forbids him to ever marry “that girl.” Based on the theory and the simple causal law that A leads to R (i.e., A ➔ R), we can now predict: John will experience reactance due to the pressure by his father which renders the option of marrying Jane more attractive (hence making a decision for marriage more likely).

### Argument 1: psychological causal explanations are incompatible with fully deterministic explanations as they are necessarily incomplete

It should be obvious that the above example is too simplistic and not even close to a clockwork-deterministic relationship or explanation. Psychological situations are always complex conditions in which several (potentially causal) factors convene (see Mackie, 1974; see also Gadenne, 2004). Researchers thus never observe causal relations between two perfectly isolated factors A and B, not even in laboratory experiments (see Argument 3).

To work properly, every theory requires boundary conditions. Applied to our example, reactance (R) does *not* occur if (1) the option in question is not perceived as personal freedom, (2) if the option is irrelevant for the person’s needs, and (3) if the social influence is perceived as competent help (Clee and Wicklund, 1980). Thus, for R to occur, we must not only propose A (patronizing pressure by the father), but a set of conditions A to D: John must also feel free to marry Jane by his own will in the first place (= B), marrying Jane must seem as important and desirable to him (= C), and John must not be convinced that his father always knew best and only wants to help (= D). Of course, good laboratory experiments thoroughly map and control such boundary conditions.

However, as Hoefer (2016) and Gadenne (2004) aptly demonstrated, this set is not nearly enough to establish a sufficient set of conditions that always and inevitably leads to reactance: We also need to assume that all else remains equal and that no disrupting influences thwart our causal prediction (i.e., A^B^C^D ➔ R). For example, it is necessary to assume that John’s phone is working and his father will not change his mind during the talk. In technical terms, we need to add a *ceteris paribus* (CP) clause to the causal explanation to make the set of conditions a cause from which the effect R, the reactance, follows with necessity.

The problem with CP clauses, however, is that they are not specified but open-ended. A perhaps infinite number of additional boundary conditions and possible confounds, ranging from the absence of psychiatric disorders to the presence of oxygen, could be introduced when trying to spell out the exact causal conditions. Causal explanations that include a CP clause are, therefore, always somewhat incomplete (Gadenne, 2004). Accordingly, if we are unable to explicate the CP condition exhaustively, it is logically possible that our psychological law will not work due to some unknown and disruptive influence that was disregarded by the explanation (Hoefer, 2016). Thus, psychologically sound (and pragmatic) causal explanations (such as A^B^C^D ➔_CP_ R) do not meet the requirements of strict clockwork determinism as R can never be inferred with inevitability.

### Argument 2: the specification of inputs and measurement of outputs will always contain variance

From the viewpoint of clockwork determinism, another problem is seen in our reactance example. To be a deterministic effect (which again serves as input for subsequent deterministic effects) our effect R should be fixed in quantity or intensity. However, we cannot identify the precise amount by which the domineering call from his father will alter John’s perception of the attractiveness of marrying Jane, even when we are fairly certain that we will observe this effect. Like most psychological theories, reactance theory does not specify fixed effects or precise functions but examines variables that are variates. If we measured the reactance-related increase in attractiveness multiple times, either repeatedly for John or across several subjects in equivalent conditions, we would always obtain a distribution of attractiveness-increase. As all psychologists know, this does not pose a problem. We could proceed to ascertain the amount of error variance, test whether there is a significant effect compared to a distribution of attractiveness values where A was not present, gauge the effect by computing intervals, and so on. That is, we use statistics to address the issue of variance in our dependent measures and test for meaningful causal effects despite the messiness of the data. However, statistics and the use of probability theory are incompatible with clockwork determinism as they assume there are multiple alternative possibilities including some level of random chance, thereby defying the principle of inevitability.

Furthermore, the problem of variance is not solely due to measurement errors or ignorance. As noted above, psychological causes are embedded in complex conditions with countless possible influences, which, if dynamically interconnected, pose firm limits to computation and prediction (see Barton, 1994; Höger, 1992; Koch, 2009; for the special case of metastability in cognitive or neurological functioning see Rabinovich et al., 2008; Freeman and Holmes, 2005). John’s experienced increase in attractiveness of the marrying-Jane option can differ by a wide margin due to variables such as his perception of the threat, proneness to reactance, or the intensity of the desire to marry Jane. Following Argument 1, all these variables would not only have to be known but determined in their exact values as well, in order to arrive at a function granting an exact and replicable attractiveness outcome. Most psychological theories, however, do not specify the exact quantities of inputs and configurations of the involved conditions. Instead, they specify meaningful (directed) psychological relationships between variables and relevant boundary conditions (e.g., “reactance outcomes increase with the growing threat to freedom”). Psychologists are therefore generally unable to predict which exact effect will occur. Accordingly, effects are labeled as quantifiable changes in the intensity of reactions or preferences on an ordinal level (i.e., using operators such as <, > or =). To suffice for determinism, however, we must not only measure but also specify and align exact responses to precise inputs (but see concluding remarks below).

### Argument 3: psychological research designs are incompatible with the requirements of clockwork determinism

Of course, one could make the point that in a perfectly controlled experiment, it would be possible to isolate discrete and repeatable responses to identical stimuli or manipulations. Indeed, as Hoefer (2016) suggests, Newtonian mechanics (and thus clockwork determinism) require two necessary ingredients to theoretically create a completely deterministic system with repeatable responses and behavior: (1) The system and its variables must be *perfectly isolated* (hence eliminating the aforementioned ceteris-paribus problem), and (2) they must be capable of imposing *identical starting conditions* to create definite testing conditions. Mathematicians have shown that a seemingly random phenomenon such as a coin toss becomes well predictable under these two conditions by a lot of precise mechanical control and adjustment (Diaconis et al., 2007). However, though this finding counts as evidence toward the validity of clockwork determinism under very specific circumstances, both requirements are by no means applicable to psychological research designs involving human subjects. It is virtually impossible to create identical, repeatable starting conditions for human subjects in within- and between-designs. Not only do all our subjects differ in various ways but also every trial and measurement irreversibly alters the subject to some extent (as psychologists are well aware). Moreover, a human being is not a metal coin that can be tied into a sophisticated mechanical apparatus (see Diaconis et al., 2007). To tightly control every aspect of a psychological situation and thus create a perfectly isolated system of a handful of variables under study (and without *any* further external influences that make CP necessary) is not only infeasible but would also encounter overwhelming problems with the ethics of experimenting.

Therefore, it is unsurprising that deterministic conditions can be emulated and observed in mechanistic systems, such as in Newtonian physics — and Newtonian physics is probably the closest scientific system to clockwork determinism. However, perfect isolation and identical starting conditions are in no (realistic) way compatible with the boundaries of psychological research, which is yet another reason why there will always be variance in psychological data (see Argument 2).

In addition, other natural sciences, such as chemistry, also rejected the existence of replicable identical starting conditions even under controlled and identical laboratory conditions. Prigogine and Stengers (1984) articulated a view of the natural world where chaos is not merely a precursor to disorder, but a fertile ground from which new forms of order and complexity can emerge. Nevertheless, in their understanding (and that of modern chaos theory as far as we are aware) uncertainty does not exclusively stem from incomplete knowledge or laws but rather represents an inherent characteristic of complex dynamic systems. This is in part due to the sensitivity of initial conditions – the notion that very small (i.e., unmeasurable) differences in a systems or an events’ initial state can lead to vastly different outcomes later on, forfeiting safe predictions even though the system itself might be working in accord with deterministic laws (also see Barton, 1994).

### Argument 4: the scale of psychological explanations is incompatible with the scale on which clockwork-deterministic explanations work

The social sciences have progressed and developed methodical access to distal factors (e.g., the impact of genes on personality, the impact of smoking on health outcomes, etc.). But again, clockwork determinism is not about probability-based relationships and sophisticated regression models of distal influences. It is about inevitable relationships and causal events that progress with necessity. Making a fully objective clockwork-deterministic prediction of the behavior of a system in, say, five years, requires taking the state of the entire world into account to arrive at 100% certainty (Hoefer, 2016). Put simply; to fully predict the next state of a system, we have to know everything about the preceding state of the system and the systems within which it is intertwined. Although the sci-fi idea of Isaac Asimov’s “psychohistory” is an entertaining literary device, a 100-percent-accurate prediction of John’s *exact* behavior in 5 years would have to include genetic configurations as well as astrophysical events with a possible impact on the solar system, life on earth, John, and so forth. Such profound access is not only physically impossible (see below) but also far out of the reach and interests of psychologists. This is precisely the reason LaPlace’s all-knowing demonic entity is just a thought experiment (one that has been falsified too, see Wolpert, 2008) but not a realistic, pragmatic assumption. Psychology and the social sciences are aptly providing models and predictions that represent effective bases for decision-making, interventions, and policies—based on statistical modeling and inductive reasoning. It is unnecessary to fulfill the requirements of clockwork determinism to further develop and hone these skills.

### Concluding remarks: why we do not need clockwork determinism, and a brief look beyond disciplinary boundaries

A critique of the fit between psychology and clockwork determinism is not synonymous with a renunciation of the scientific method or an ontological refutation of classic clockwork determinism. Lawfulness is not necessarily bound to strict causal relations, and fully deterministic causation is only one possible mode of lawful causality (Bunge, 1979). Lawfulness can also be found in statistical events that contain chance and uncertainty. Thus, stochastic hypotheses can be experimentally tested and corroborated (or falsified) with data. For example, we may not be able to precisely determine what the next throw of dice will yield, but we can establish with certainty that for a large number of dice throws an even distribution of 1 s to 6 s is more likely than a distribution of only 1 s. Hence, by using statistics and actively working with multiple possibilities and probability theory we can still derive meaningful and accurate conclusions.

Furthermore, our issue is agnostic to the reality of determinism. We present arguments that psychology does not, and never will, encompass laws that are not limited by the ceteris-paribus addition, or indeed by uncertainty. According to the arguments herein, psychology will always yield somewhat incomplete explanations rather than strict and all-encompassing laws. Therefore, it would be illogical to hold the claim that psychology is a science that should search for fully deterministic, strict laws. Psychology research cannot permanently meet this claim. However, refuting this claim on the grounds of arguments 1 to 4 does not include evidence for the conclusion that strict deterministic laws do not exist. We merely propose an incompatibility between psychological science and the requirements of clockwork determinism, while the latter still might exist. Refuting classic determinism itself calls for different arguments.

Finally, it is helpful to know that physics and other natural sciences already show the productive value of acknowledging firm natural limits to the determinability and predictability of events, whether they are due to the nature of nonlinear dynamic systems (see Barton, 1994; Höger, 1992) or quantum principles (Heisenberg, 1927). A significant portion of natural science departed from clockwork determinism long ago and thrived. Put differently, if physics can accept the finding that an observable entity is neither a wave nor a particle but both simultaneously (Einstein and Infeld, 1967), psychology might also remain open to multiple outcomes of the same system in the same experimental conditions. Indeed, Baumeister (in press) recently contended that clockwork determinism is an obsolete worldview, rooted in antiquity but flourishing best in the Enlightenment age (the 18th century), and that 20th century advances in physics (relativity, quantum mechanics), chemistry, and philosophy (phenomenology, existentialism) rendered it untenable.

## Conclusion

Clockwork determinism might represent a powerful tool to reduce uncertainty, because it postulates inevitabilities by assuming them for any relationship between events. Uncertainty is ubiquitous (Doob, 1988) and therefore represents a challenge for everyone. For example, by arising from competing and thus conflicting perceptual and behavioral affordances, uncertainty can create the experience of psychological entropy, thereby posing a critical adaptive challenge that has to be managed (Hirsh et al., 2012, p. 304–306). Consequently, there is a broad preference for minimizing and avoiding uncertainty, despite its sometimes-positive effects (Alquist and Baumeister, 2023). Starting a scientific career and tackling big questions in an ever-changing, competitive environment possibly constitutes an entropic challenge that enhances the appeal of deterministic doctrines or beliefs. This hypothesis might explain why it was found that junior psychologists tend to endorse determinism as a doctrine more than senior scientists (see Baumeister and Lau, 2024). There is also reason to be skeptical about the actual effectiveness of deterministic beliefs to cope with uncertainty. However, we are certain that research careers that focus on finding deterministic laws, where logically none can be formulated, are problematic. As we have shown, the established scientific practice of experimental quantitative research is incompatible with the requirements of clockwork determinism. When suspending strict determinism as a doctrine, this practice can therefore be retained without losing any of the successful or promising research themes that acknowledge the vast range of possibilities in mental and social processes.

## Footnotes

^1^We cordially thank Volker Gadenne for discussion and helpful insights on these points.
